# Multivalent Neuroprotective Activity of *Elettaria cardamomum* (Cardamom) and *Foeniculum vulgare* (Fennel) in H_2_O_2_-Induced Oxidative Stress in SH-SY5Y Cells and Acellular Assays

**DOI:** 10.3390/ph18010002

**Published:** 2024-12-24

**Authors:** Himadri Sharma, Hyewon Yang, Niti Sharma, Seong Soo A. An

**Affiliations:** Department of Bionano Technology, Gachon Bionano Research Institute, Gachon University, 1342 Seongnam-daero, Sujeong-gu, Seongnam-si 461-701, Gyeonggi-do, Republic of Korea; himadri@gachon.ac.kr (H.S.);

**Keywords:** cardamom, fennel, anti-acetylcholine esterase activity, anti-Aβ fibrillization/oligomerization, anethol, α-terpinyl acetate, neuroprotection, oxidative stress

## Abstract

Background: *Elettaria cardamomum* (Cardamom) and *Foeniculum vulgare* (Fennel) are well-known spices and are also used as natural mouth fresheners. This study was performed to evaluate their neuroprotective ability based on certain acellular and cellular assays. Methods: Hexane and ethyl acetate extracts were prepared using cardamom and fennel seeds. GC/MS was performed for the identification of important bioactive compounds. Cell-based assays were performed using SH-SY5Y cells. Hydrogen peroxide was used for the induction of oxidative stress, and evaluation was done based on neuroprotection, reduced reactive oxygen species, and restoration of mitochondrial membrane potential (MMP). Additionally, anti-Aβ fibrillization/oligomerization activities were also analyzed along with anti-acetylcholinesterase activity. Results: α-Terpinyl acetate and anethol were identified as major phytocompounds in cardamom and fennel, respectively. Cardamom extracts and α-terpinyl acetate were more potent acetylcholinesterase (AChE) inhibitors than fennel extracts and anethol [IC_50_ cardamom extracts, 130–150 μg/mL; α-terpinyl acetate, 61.87 μg/mL; anethol, 374.2 μg/mL; fennel extracts, >1 mg/mL] and showed mixed-type inhibition. Only the extracts displayed potent anti-Aβ fibrilization activity (>50%). Anethol showed potent anti-Aβ oligomerization activity (>50%), followed by α-terpinyl acetate and fennel-H (~36%). The neuroprotective potential of the spice extracts/phytochemicals was evaluated in SH-SY5Y cells by using H_2_O_2_-induced oxidative stress. Cardamom-EA displayed the best neuroprotection (0.01 to 30 μg/mL). No neuroprotection was observed by α-terpinyl acetate and anethol. Cardamom extracts and fennel-H restored the normal reactive oxygen species (ROS) levels at 30 µg/mL and 1 µg/mL, respectively. Conclusion: Overall, the extracts provided better neuroprotection than the pure compounds in cellular assays and displayed strong anti-Aβ fibrilization activity.

## 1. Introduction

Neurodegenerative diseases (NDs) are caused by progressive degradation/loss of neurons. These changes may occur over the years, leading to cognitive and functional decline. The most common ND is Alzheimer’s disease (AD), which is the fourth leading reason for disability in the elderly population [1]. Most of the NDs share common interrelated pathological events like increased oxidative stress (OS) and neuroinflammation. Exogenous and endogenous sources may cause an increase in cellular oxidative stress [2], enhancing the deposition of oligomeric amyloid beta (Aβ), which in turn triggers tau hyperphosphorylation and the formation and build-up of neurofibrillary tangles (NFTs) in neurons [3]. OS generation initiates a cycle of superoxide radicals (O2 •–), reactive oxygen and nitrogen species (ROS, RNS), and peroxynitrite (ONOO−) production that disrupts the normal functioning of proteins, enzymes, and ion channels [4,5]. In cellular models, hydrogen peroxide (H_2_O_2_) exposure is widely used to induce oxidative stress in the cell. The OH radicals generated by H_2_O_2_ and Fe^2+^ ions (Fenton’s reaction) increase oxidative injury to the cells [6]. Under healthy conditions, when the redox reactions are balanced, the inflammatory system acts as the defense mechanism, but under redox imbalance, as in NDs, neuroinflammation is increased in the CNS [7]. Over the years, many signaling molecules and neurotransmitters have been identified that can act as therapeutic targets. Several phytocompounds have been extracted and used against these targets [8]. The market value for medicinal plants, according to a 2019 report, was estimated at approx. 84.5 billion USD, which increased to 164.22 billion USD in 2023 and is anticipated to increase to 879.65 billion by 2032 [9,10]. Most NDs comprise multifactorial symptoms that affect the patients differently; this increases the need to have a cost-effective, safe, and potent multitargeted drug.

Cardamom (*Elettaria cardamomum* (L.) Maton) belongs to the Zingiberaceae family, and its aromatic seeds are used as a mouth freshener, flavoring agent, and spice in different cuisines. It is mostly grown in the moist forests of southern India, Sri Lanka, Guatemala, and Indonesia. Cardamom is a perennial herbaceous plant with different phytocompounds such as flavonoids, tannins, phenols, terpenoids, sterols, and proteins [11,12]. The supercritical Cardamom (fruit and seeds) extract also exhibits various pharmacological properties such as anti-inflammatory, antioxidant, antimicrobial, and anticancer [13]. Traditionally, cardamom seeds have been used to treat toothaches, cataracts, asthma, and digestive disorders [14]. Cardamom oil inhibited acetylcholinesterase (AChE), reduced oxidative damage, and exerted anti-anxiety effects in aluminum-induced neurotoxicity in rats [15]. It also exerted an antidepressant effect by stimulating serotonergic, dopaminergic, and noradrenergic systems and increased brain-derived neurotrophic factor (BDNF) in the rat brain cortex and hippocampus [16]. Cardamom fruit extract (petroleum ether) reportedly decreased amyloid beta (Aβ) and phosphorylated tau (p-tau) levels and lowered the expression of AChE and caspase-3 activity in the rat hippocampus [17]. The ethanolic seed extract of cardamom and its phytocompound (α-terpinyl acetate) displayed anti-AChE and anti-butyrylcholine esterase (BChE) activities. They also reduced hydrogen peroxide (H_2_O_2_)-stress in rat pheochromocytoma (PC12) cells, reduced Aβ-induced neurotoxicity, and displayed antioxidant and anti-amyloidogenic properties [18].

Fennel (*Foeniculum vulgare* Mill.; family Apiaceae) is a perennial herb that is native to southern Europe along the Mediterranean Sea and is also grown in parts of Asia. It is used in traditional medicine to treat digestive, reproductive, respiratory, and endocrine systems-related ailments [19]. It has antimicrobial, antioxidant, anticancer, hepatoprotective, chemopreventive, hypoglycemic, and estrogenic activities [19,20,21,22]. The inhalation of fennel essential oil ameliorated Aβ1-42-induced depression and anxiety in rats [23]. The nootropic, anti-AChE, anti-inflammatory, and neuroprotective nature of the whole plant of fennel (methanolic extract) have been reported in mice [24]. Anethol (a phytocompound of fennel) reportedly improved cognitive function (250 mg/kg, i.g.) in rotenone-induced cognitive deficit rats by decreasing lipid peroxidation (MDA) and increasing the number of surviving neurons in the hippocampus [25] and expression of α-synuclein [26]. Fennel and anethol have been reported to prevent stress-induced neurological disorders in social isolation-induced behavioral deficit rats by improving memory and learning [27]. Fennel seed extracts (ethanol, methanol, hexane) lowered neuronal toxicity in lead-induced neurotoxicity in mice by improving oxidative stress markers and normalizing amyloid precursor protein (APP) levels [28].

This research was carried out using human neuroblastoma cells (SH-SY5Y) to gain a deeper insight into how the nonpolar (hexane and ethyl acetate) extracts of cardamom and fennel and their major phytochemicals regulate the effects of H_2_O_2_-induced oxidative stress-related neurodegeneration and its associated disease progression. Furthermore, the extracts/phytocompounds were also assessed for their anti-AChE and anti-Aβ oligomerization/fibrillation activities.

## 2. Results

### 2.1. GC-MS Analysis

**Figure 1:**
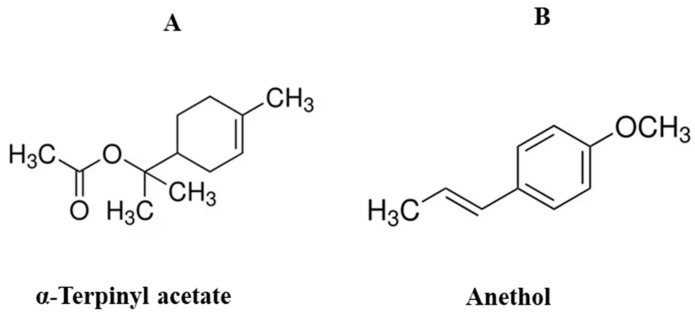
The main phytocompound in (**A**) cardamom and (**B**) fennel extracts.

The GC-MS analysis of the hexane and ethyl acetate fractions of cardamom and fennel was carried out. The peaks were identified by comparing their retention time, peak area and height%, and mass spectral fragmentation with those of the known compounds in the NIST library (Supplementary Figure S1). The major peak of α-terpinyl acetate (12.43 min) was observed in cardamom extracts (54.89% in cardamom-H and 64.83% in cardamom-EA). Anethol was the major component in fennel extracts (51.9% in fennel-H and 31.87% in fennel-EA) at 11.58 min. The structural formula for these major phytocompounds has been given below (Figure 1).

### 2.2. Phytochemical Assessment

Colorimetric assays measured the phenolics and flavonoids in the extracts. The TPC was calculated from the regression equation of the calibration curve (R^2^ = 0.995, y = 0.046x + 0.0059) and expressed in gallic acid equivalent (GAE) as milligrams per gram of the extract (mg GAE/g extract). The TPC was higher in fennel (26.91 ± 7.31 mg GAE/g in fennel-H; 32.43 ± 0.08 mg GAE/g in fennel-EA) than cardamom (7.95 ± 0.94 mg GAE/g in cardamom-H; 4.49 ± 1.01 mg GAE/g in cardamom-EA). The TFC was determined using the regression equation of the calibration curve (R^2^ = 0.99, y = 0.0428x + 0.0103), reported in quercetin equivalent (QE) as milligrams per gram of the extract (mg QE/g extract). The TFC was highest in cardamom-EA (42.19 ± 0.94 mg QE/g), followed by fennel-EA (13.63 ± 0.50 mg QE/g), fennel-H (10.82 ± 0.80 mg QE/g), and cardamom-H (4.49 ± 1.01 mg QE/g). The antioxidant activity of the pure compounds was also estimated using FRAP, DPPH, and ABTS methods. Fennel-EA displayed the best antioxidant activity as compared to others in all these assays (Table 1).

### 2.3. Anti-Acetyl Cholinesterase Activity

**Figure 2:**
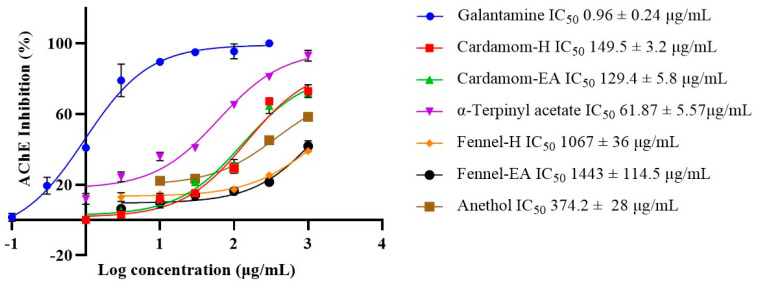
IC_50_ curves of cardamom and fennel extracts and their major phytocompounds (α-terpinyl acetate and anethol) with inhibitor control against AChE (*Electrophorus)*. The IC_50_ values were computed using GraphPad Prism 10.2. All data are expressed as mean ± SD (*n* = 3).

Acetylcholinesterase (AChE) inhibitors impede the normal breakdown of acetylcholine (ACh). AChE is one of the most studied targets for AD. Donepezil, rivastigmine, and galantamine are the three United States Food and Drug Administration (USFDA)-approved AChE inhibitors to treat AD treatment. Hence, the samples were tested for their anti-AChE effects, and the IC_50_ values were determined with galantamine as the reference inhibitor. Cardamom extracts and α-terpinyl acetate were more potent AChE inhibitors than fennel extracts and anethol. The IC_50_ value of cardamom-H (149.5 ± 3.2 μg/mL) was comparable to cardamom-EA (129.4 ± 5.8 μg/mL) extract. Fennel-EA showed a lower value (IC_50_ 1443 ± 114.5 μg/mL) compared to fennel-H (IC_50_ 1067 ± 36 μg/mL) and anethol (IC_50_ 374.2 ± 5.8 μg/mL: 2.52 mM). α-Terpinyl acetate displayed the lowest IC_50_ value (61.87 ± 5.57 μg/mL: 51.71 μM) than the others. The IC_50_ value of galantamine was 0.96 ± 0.24 μg/mL (3.34 μM) (Figure 2).

**Figure 3:**
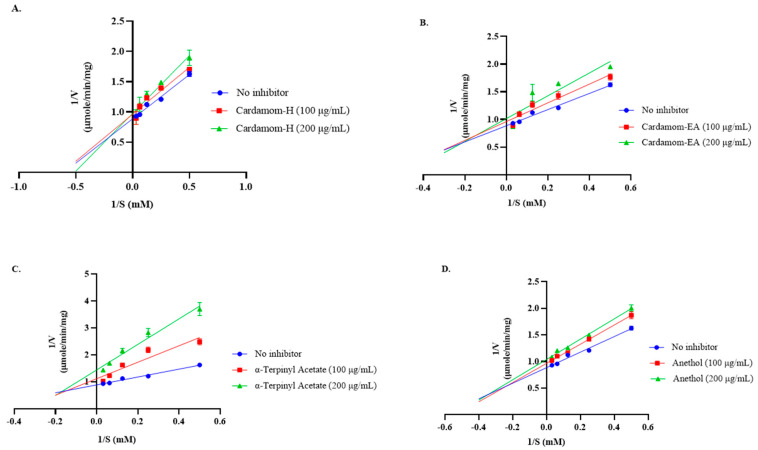
Lineweaver-Burk plots of AChE in the presence of 100 μg/mL and 200 μg/mL of (**A**) cardamom-H, (**B**) cardamom-EA, (**C**) α-terpinyl acetate, and (**D**) anethol. The graphs were plotted using GraphPad Prism 10.2. Abbreviations: V: Velocity of enzyme-catalyzed reaction; S: Substrate.

For the kinetic analysis, fennel extracts were omitted as they displayed IC_50_ > 1 mg/mL. The Lineweaver-Burk plots (Figure 3) indicated mixed-type inhibition by cardamom extracts and pure compounds. In mixed inhibition, the inhibitor may bind to the enzyme (E) as displayed by the competitive inhibitor or with the enzyme-substrate (ES) complex as shown by the uncompetitive inhibitor. The kinetic data has been represented in Table 2.

In mixed inhibition, the inhibitor may bind to the enzyme (E) as displayed by the competitive inhibitor or with the enzyme-substrate (ES) complex as shown by the uncompetitive inhibitor. The kinetic data has been represented in Table 2.

### 2.4. Aβ-Fibrilization and Oligomerization Inhibition

**Figure 4:**
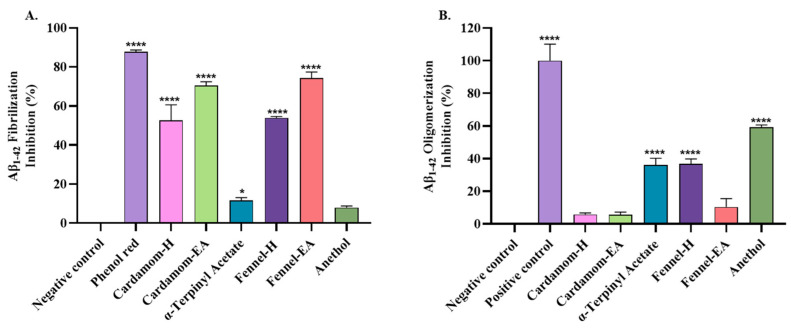
Aβ oligomerization and fibrilization inhibition displayed by cardamom, fennel extracts, and pure compounds. (**A**) ThT anti-fibrilization assay. (**B**) Aβ oligomerization inhibition. All data were expressed as mean ± SD (*n* = 3). A significant difference * (*p* < 0.05) and **** (*p* < 0.0001) using the two-way ANOVA (**A**) and one-way ANOVA (**B**) followed by Dunnett’s post hoc test was observed in samples vs. negative control.

Aβ-fibrilization inhibition by the samples was monitored using a benzothiazole-based fluorophore (ThT). ThT is a widely used fluorescent dye to monitor amyloid fibril assembly, as the interaction of ThT with fibril results in a significant change in the excitation peak (from 385 nm to 450 nm), which is easy to detect [29]. For the ThT assay, 500 μg/mL extracts/pure compounds were tested for anti-fibril potential, using phenol red as the reference. The findings showed statistical significance (**** *p* < 0.0001) when compared to the negative control (Buffer + Aβ) (Figure 4A). The extracts displayed similar anti-fibrilization activity, with the ethyl acetate extract performing better than the hexane extract. The inhibition shown by the extracts was 52.60 ± 8.02% (cardamom-H), 70.57 ± 1.88% (cardamom-EA), 53.96 ± 0.64% (fennel-H), and 74.25 ± 3.20% (fennel-EA). On the other hand, the pure compounds α-terpinyl acetate and anethol merely inhibited fibrilization with 11.65 ± 1.42% (* *p* < 0.5) and 7.94 ± 0.84% (non-significant; ns), respectively. The positive control showed 87.71 ± 0.95% (**** *p* < 0.0001) inhibitory activity higher than the previously reported value of 71.34 ± 2.9% [5].

The MDS assay was used to assess the Aβ oligomerization inhibition activity of the samples. From the results (Figure 4B), anethol significantly reduced Aβ1-42 oligomerization tendency with 59.37 ± 1.25% (**** *p* < 0.0001) oligomerization inhibition. Fennel-H displayed 36.73 ± 3.11% inhibition similar to α-terpinyl acetate 36.27 ± 4%; **** *p* < 0.0001. On the other hand, weak and non-significant inhibition was registered for fennel-EA (10.39 ± 5.14%), cardamom-H (5.86 ± 0.89%), and cardamom-EA (5.64 ± 1.32%).

### 2.5. Cytotoxicity Evaluation

**Figure 5:**
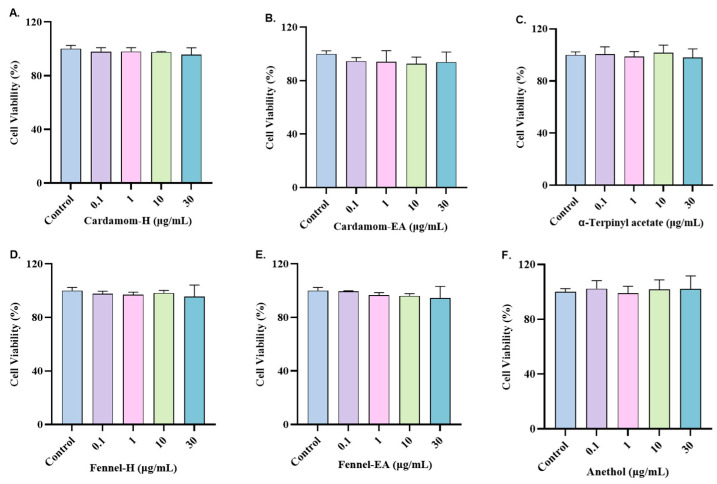
Cytotoxicity assay of (**A**) cardamom-H, (**B**) cardamom-EA, (**C**) α-terpinyl acetate, (**D**) fennel-H, (**E**) fennel-EA, and (**F**) anethol on the SH-SY5Y cells. The cell viability was reported as the percentage of the control group (100%). All data were presented as mean ± SD (*n* = 3). No significant difference was observed using one-way ANOVA followed by Dunnett’s post hoc in the % of cell viability in the treated vs. the untreated control cells.

The cytotoxic effect of cardamom and fennel extracts and the major phytocompounds were assessed on the neuroblastoma cell line (SH-SY5Y). The cells were pre-treated with different concentrations of the samples (0.1, 1, 10, 30 μg/mL) for 24 h, and the cell viability was measured at 405 nm by adding WST-8 dye. The cell viability was over 94%, and no significant cytotoxicity was seen in the samples at the tested concentrations (Figure 5).

### 2.6. Neuroprotective Activity

The neuroprotective effect of the samples was studied by the induction of oxidative stress in the SH-SY5Y cells by H_2_O_2_. Nearly 50% cell death was observed with 100 µM H_2_O_2_, and hence it was used to induce oxidative stress in the cells pre-incubated with the samples for 24 h.

**Figure 6:**
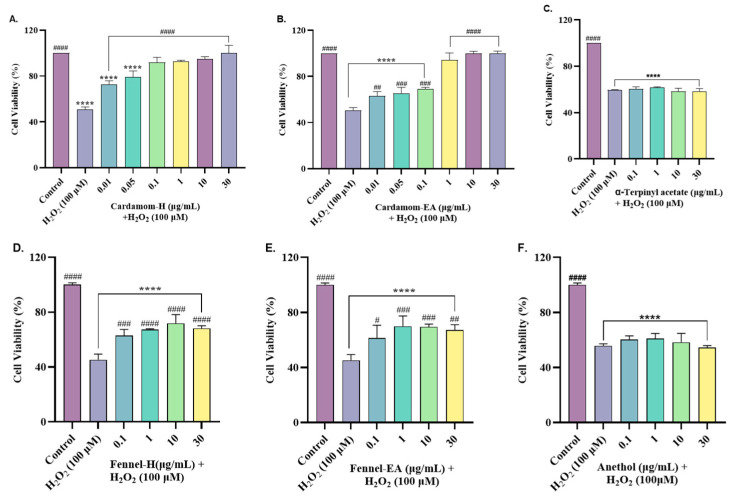
Neuroprotective effects of various concentrations of (**A**) cardamom-H, (**B**) cardamom-EA, (**C**) α-terpinyl acetate, (**D**) fennel-H, (**E**) fennel-EA, and (**F**) anethol on H_2_O_2_-induced SH-SY5Y cells. The results indicate % cell viability vs. the control cells. All data were presented as mean ± SD (*n* = 3). Using one-way ANOVA followed by Dunnett’s test, a significant difference ^#^ (*p* < 0.05), ^##^ (*p* < 0.01), ^###^ (*p* < 0.001), and ****^/####^ (*p* < 0.0001) was observed in the % of cell viability vs. untreated control cells (*) and H_2_O_2_-treated cells (^#^).

Cardamom-H displayed a significant increase (#### *p* < 0.0001) in the cell viability at 0.1 µg/mL (91.98 ± 4.32%); 1 µg/mL (92.61 ± 4.32%); 10 µg/mL (94.89 ± 2.01%); and 30 µg/mL (100 ± 6.80%) compared to the H_2_O_2_ control (Figure 6A). As the neuroprotection was over 90% at 0.1 µg/mL, we tried two further lower concentrations. A significant dose-dependent increase in cell viability was seen from 0.01 to 0.1 µg/mL, after which it remained nearly constant. At 0.01 µg/mL (72.96 ± 2.85%) and 0.05 µg/mL (79.44 ± 5.04%), the increase in cell viability was significant (#### *p* < 0.0001) compared to the H_2_O_2_ control. Cardamom-EA also exerted neuroprotection by increasing cell viability at 0.01 µg/mL (63.03 ± 3.81%, ## *p* < 0.01); 0.05 µg/mL (65.42 ± 4.97%, ### *p* < 0.001); 0.1 µg/mL (69.06 ± 1.46%, ### *p* < 0.001); and 1 µg/mL (94.38 ± 6.08%, #### *p* < 0.0001); 10 µg/mL (100 ± 1.95%, #### *p* < 0.0001); and 30 µg/mL (100 ± 2.04%, #### *p* < 0.0001) compared to the H_2_O_2_control (Figure 6B). Fennel extracts showed lower neuroprotection compared to the cardamom extracts. Fennel-H showed a significant (#### *p* < 0.0001) dose-dependent increase in cell viability from 0.1 to 10 µg/mL (#### *p* < 0.0001), after which it decreased slightly at 30 µg/mL (Figure 6D). In the case of fennel-EA, an increase in cell viability was observed at 0. 1 µg/mL (61.38 ± 9.38%, # *p* < 0.05); 1 µg/mL (69.97 ± 0.58%, ### *p* < 0.001); 10 µg/mL (69.58 ± 1.99%, ### *p* < 0.001); and 30 µg/mL (67.26 ± 3.85%, ## *p* < 0.01) (Figure 6E). In our study, α-terpinyl acetate (Figure 6C) and anethol (Figure 6F) failed to show neuroprotection at the tested concentrations. Among the extracts, cardamom-EA displayed the best neuroprotection, followed by cardamom-H and fennel extracts.

### 2.7. Effect on Intracellular Reactive Oxygen Species (ROS) Generation

**Figure 7:**
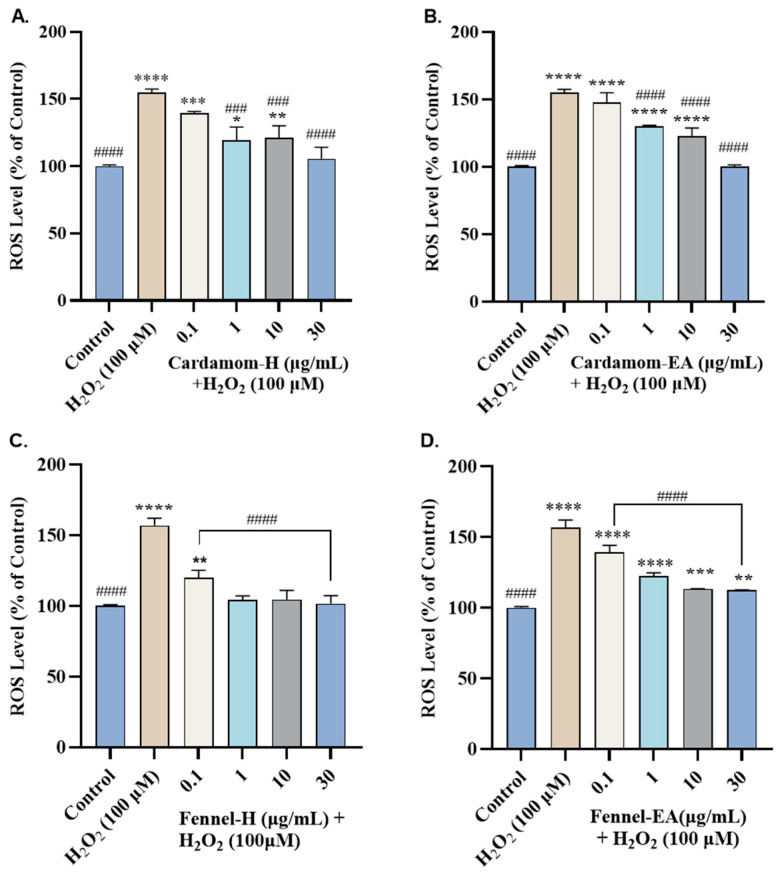
Effects of various concentrations of (**A**) cardamom-H, (**B**) cardamom-EA, (**C**) fennel-H, and (**D**) fennel-EA on H_2_O_2_-induced ROS generation in neuroblastoma SH-SY5Y cells. The results indicate % cell viability vs. the control cells. All data are presented as mean ± SD (*n* = 3). Using one-way ANOVA followed by Dunnett’s test, a significant difference * (*p* < 0.05), ** (*p* < 0.01), ***^/###^ (*p* < 0.001), and ****^/####^ (*p* < 0.0001) was observed in the% of cell viability vs. untreated control cells (*) and H_2_O_2_-treated cells (^#^).

The fluorescent dye H2DCFDA was used to track ROS production in the cells, as it is transformed into DCF by ROS. In our study, 155.91 ± 1.48% ROS was generated by 100 µM H_2_O_2_-treated cells compared to the untreated control cells (100%). Pretreatment of the cells with cardamom-H decreased ROS production at 0.1 µg/mL (139.51 ± 1.19%, ns), 1 µg/mL (119.05 ± 9.94%, #### *p* < 0.0001), 10 µg/mL (120.98 ± 9.11% at, ### *p* < 0.001), and 30 µg/mL (105.50 ± 8.51%, #### *p* < 0.0001) compared to the H_2_O_2_-treated cells (Figure 7A). Cardamom-EA treatment reduced the ROS in a dose-dependent manner (147.84 ± 7.38% at 0.1 µg/mL, ns; 130.07 ± 0.63% at 1 µg/mL, ### *p* < 0.001; 122.70 ± 6.09% at 10 µg/mL, #### *p* < 0.0001; and 100.01 ± 1.45% at 30 µg/mL, #### *p* < 0.0001) compared to the H_2_O_2_-treated cells (Figure 7B). A significant (#### *p* < 0.0001) reduction in ROS was seen in the fennel extracts. Fennel-H restored the ROS levels to the control at 1 µg/mL (104.26 ± 2.79%, #### *p* < 0.0001) and remained constant up to 30 µg/mL (Figure 7C). On the other hand, fennel-EA showed a significant (#### *p* < 0.0001) dose-dependent decrease (139.16 ± 5.00% at 0.1 µg/mL, 122.34 ± 2.33% at 1 µg/mL, 113.36 ± 0.28% at 10 µg/mL, and 112.44 ± 0.37% at 30 µg/mL) (Figure 7D). From the results, fennel-H displayed better ROS scavenging potential than the cardamom extracts.

### 2.8. Effect on Mitochondrial Membrane Potential (MMP, ΔΨm)

The mitochondrial membrane potential (ΔΨm) was measured using TMRE dye. The depolarized membrane has a lower ΔΨm due to the inability to trap the dye effectively. We observed ~50% reduction in the ΔΨm in the untreated cells at 200 μM H_2_O_2_; hence, this concentration was used for the assay.

**Figure 8:**
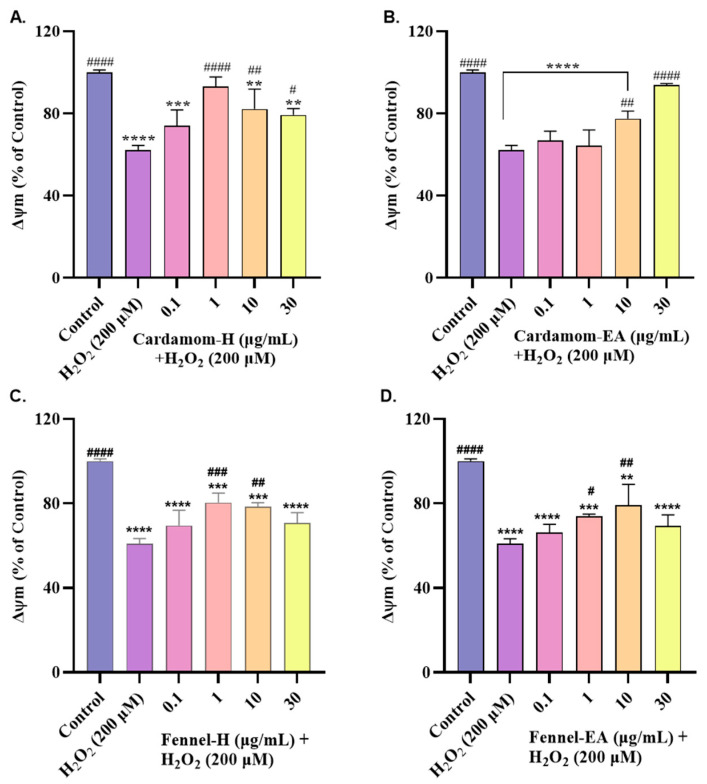
Effects of various concentrations of (**A**) cardamom-H, (**B**) cardamom-EA, (**C**) fennel-H, and (**D**) fennel-EA on mitochondrial membrane potential (ΔΨm) in H_2_O_2_-induced SH-SY5Y cells. The results indicate % cell viability vs. the control cells. All data are presented as mean ± SD (*n* = 3). Using one-way ANOVA followed by Dunnett’s test, a significant difference ^#^ (*p* < 0.05), **/^##^ (*p* < 0.01), ***/^###^ (*p* < 0.001), and ****^/####^ (*p* < 0.0001) was observed in the % of cell viability vs. untreated control cells (*) and H_2_O_2_-treated cells (^#^).

Pre-incubation with the extracts increased MMP to variable extents. In cardamom-H, MMP increased from 0.1 µg/mL (73.95 ± 7.77%, ns) to 1 µg/mL (93.18 ± 4.53%, #### *p* < 0.0001) and decreased afterward (82.13 ± 9.75% at 10 µg/mL, ## *p* < 0.01; and 79.28 ± 3.04% at 30 µg/mL, # *p* < 0.05) (Figure 8A). Cardamom-EA increased the MMP from 10 µg/mL (77.41 ± 3.77%, ## *p* < 0.01) to 30 µg/mL (93.89 ± 0.65%, #### *p* < 0.0001) while no effect was seen at the lower concentrations (Figure 8B). Fennel-H showed a dose-dependent increase in MMP from 0.1 µg/mL (69.53 ± 7.23%, ns) to 1 µg/mL (80.42 ± 4.53%, ### *p* < 0.001), after which it remained almost constant at 10 µg/mL (78.47 ± 1.91%, ## *p* < 0.01) and declined afterward at 30 µg/mL (70.73 ± 4.94%, ns) (Figure 8C). Fennel-EA treatment increased the MMP in a dose-dependent manner from 0.1 µg/mL to 10 µg/mL (79.13 ± 9.94%, ## *p* < 0.01), after which it declined at 30 µg/mL (69.32 ± 5.34%, ns) (Figure 8D).

## 3. Discussion

In the present study, we have evaluated the neuroprotective potential of cardamom and fennel extracts and their main phytocompounds in SH-SY5Yneuroblastoma cell lines. We measured their TPC, TFC, and antioxidant activities. In our study, the phenolic content of fennel-EA was highest and exhibited the best antioxidant activity among all other extracts. Phenolic compounds function as antioxidants by interacting with various free radicals. The mechanisms of antioxidant actions can include hydrogen atom transfer, single electron transfer, sequential proton loss electron transfer, and chelation of transition metals [30,31]. A positive correlation has been reported between TPC and antioxidant activity in the literature [5]. Previously, a TPC of 13.09 ± 0.95 mg GAE/g was reported for cardamom-EA [32], similar to our result. A TPC of 24.2 ± 0.29 mg GA/g and 30.3 ± 0.7 mg GAE/g have been reported in aqueous extracts of cardamom and fennel, respectively [33]. The TPC (fennel 8.31 ± 0.03 mg GAE/g; cardamom 3.30 ± 0.32 mg GAE/g) and TFC (fennel 0.19 ± 1.8 mg QE/g; cardamom 0.13 ± 0.04 mg QE/g) were also evaluated in fennel and cardamom hydroalcoholic (1:1) extracts. In the same study, no significant difference was reported for antioxidant activity in the whole spice hydroalcoholic extract of cardamom and fennel [34]. For fennel seeds (aqueous, acetone, and ethanolic extract), the FRAP values varied from 608–1179 µM Fe^2+^/g, while the DPPH assay results displayed 24–78% activity [35]. However, results similar to ours were reported for the DPPH activity of α-terpinyl acetate [18] and ABTS activity of anethol [36]. The spice extracts consist of an intricate blend of biologically active substances. The levels of phenolic acids and flavonoids are influenced by the variety, soil, and climatic conditions. The antioxidant properties of spices differ based on the extraction method used.

The GC-MS analysis identified α-terpinyl acetate and anethol as the major peaks in cardamom and fennel, respectively. α-Terpinyl acetate (C_12_H_20_O_2_; molecular weight: 196.28 g/mol) is a p-menthane monoterpene ester detected in cardamom extracts. Previously, α-terpinyl acetate was identified as a major component in cardamom seeds by molecularly imprinted polymer-quartz crystal microbalance (MIP-QCM) sensors [37], low-temperature extraction [38], hydro-distillation using Clevenger followed by GC/MS [39] and HPTLC [40], and instant controlled pressure drop technology coupled with sonication [41].

It is approved as safe for its use as a food-flavoring agent by the Flavor and Extract Manufacturers Association (FEMA) and the EU Food Improvement Agency (FIA) [42,43]. It is commercially employed as an aroma component in cosmetics, air purifiers, and detergents and is also used in the bakery for flavor enhancement [44]. α-Terpinyl acetate has been reported to be a possible ligand in AD [18] and has antioxidant, anti-inflammatory [45], and antimicrobial activities [44]. Anethol (C_10_H_12_O; Molecular weight: 148.20 g/mol), a derivative of allylbenzene, was the most abundant compound in fennel extracts. Anethol was identified as a major essential oil in fennel by GC-MS and 1H-NMR spectroscopy [46], hexane seed extract by HPLC [47], methanolic extract by RP-HPLC [48], and ethanolic seed extract by GC/MS [49]. It is generally recognized as safe (GRAS) [50] to be used as a flavoring agent in food. Anethol is around 13 times sweeter than natural sugar; therefore, it is used in confectionery and drinks. It has potent antioxidant, antimicrobial, antifungal, and insecticidal activities [51,52,53]. The methoxy (-OCH3) group in anethol might be responsible for the better antioxidant activity of fennel extracts [54].

Acetylcholinesterase (AChE; E.C.3.1.1.7) is a cholinergic enzyme found mainly at the postsynaptic neuromuscular junctions, breaking down ACh, a vital neurotransmitter. In AD there is a decrease in the amount of ACh at the synaptic junction, so it is beneficial to block AChE to uphold proper levels of ACh. AChE inhibition may help decrease Aβ plaques and neurofibrillary tangles (NFT) formation [55]. In our study, cardamom extracts and α-terpinyl acetate were more potent AChE inhibitors than fennel extracts and anethol. α-Terpinyl acetate displayed the lowest IC_50_ value (61.87 ± 5.57 μg/mL: 51.71 μM) than the others. Previous research reported a similar IC_50_ value (54.72 ± 10.03 μM) for AChE inhibition by α-terpinyl acetate [18].

From the kinetic parameters, Km (the concentration of the substrate that permits the enzyme to achieve half Vmax) varied and Vmax (the maximal reaction velocity when the enzyme is saturated with its substrate) decreased compared to no inhibitor, indicating the mixed-type AChE inhibition. Thus, cardamom extracts, α-terpinyl acetate, and anethol can bind to the free enzyme € or the ES (enzyme substrate) complex. The ethanolic and aqueous extracts, essential oil, and a polyherbal formulation containing cardamom have been reported to inhibit AChE [56,57]. The oral administration of cardamom oil (100 and 200 mg/kg body weight) provided neuroprotection in the AlCl3-induced neurotoxic rat model by inhibiting AChE and reducing oxidative damage [15]. The methanolic and ethanolic extracts of fennel have also effectively inhibited AChE and improved cognition in animal models [24,28,58]. α-Terpinyl acetate was reported as a competitive inhibition of AChE. Docking studies revealed that it interacted with human AChE residues at the catalytic site (Ser203, His447, and Glu334), acyl-binding pocket (Phe295 and Phe297), peripheral anionic site (Tyr124), quaternary ammonium binding site (Trp86), and oxyanion hole (Gly120 and Gly121) [18]. However, in our study, mixed-type inhibition was shown by α-terpinyl acetate. The alteration in the type of inhibition might be due to the different enzyme sources used.

Aβ fibrils are the primary constituent of amyloid plaques found in AD brains. These fibrils exhibit different molecular structures (polymorphs), leading to differences in AD severity and clinical symptoms [59]. Anti-fibrilization inhibitors might interact with fibril-forming monomers, block the growing fibril tips, or attach to oligomers’ surfaces to hinder nucleation [60]. Moreover, compounds that interact with the central region of Aβ prevent the formation of oligomers [61]. Previously, α-terpinyl acetate inhibited protein aggregation by 51.08% in the ThT assay [18] and destabilized pre-formed fibrils [62]. Docking studies revealed that α-terpinyl acetate interacted through hydrogen bonding with Try63 and by hydrophobic interactions with the other residues (Arg61, Trp62, Ala107, Trp108, Val109, Arg112) in the amyloidogenic region [18]. In addition, the β-amyloid inhibitory activity of cardamom [15] and fennel [28,63] has been reported. In our study, cardamom and fennel extracts displayed more potential anti-fibrilization activity (50–70%) than the pure compounds. Similar results were reported earlier, where 1,8-cineole-rich cardamom extract showed better anti-fibrilization potential than pure 1,8-cineole (eucalyptol) [64]. Fennel also has this compound, and fennel supplementation was reported to reduce serum Aβ concentration by 21.53% [63]. Elevated amyloid precursor protein (APP) levels increase Aβ production. The ethanolic seed extract of fennel (200 mg/kg/day) normalized the expression of APP in the lead-induced brain neurotoxicity mice model [28]. In addition, eugenol in fennel also displayed anti-fibrilization activity in our previous study [5]. It is speculated that aromatic compounds in extracts disrupt the protein’s β-sheet structure via π-stacking or hydrophobic interaction, hindering Aβ fibril formation. The lower anti-fibrilization activity of pure compounds compared to the extracts indicates potential synergistic or additive effects of phytochemicals in the extracts for anti-amyloidogenic properties [5]. In the Aβ-oligomerization inhibition study, anethol performed better than the others, which might be due to the methoxy group that could potentially enhance the anethol’s interaction with Aβ oligomers [65]. The higher levels of anethol in fennel-H could be the reason for the better anti-oligomerization potential of fennel-H than others.

SH-SY5Y cells were exposed to H_2_O_2_ to induce oxidative stress. Highly reactive OH radicals are generated by Fenton’s reaction between H_2_O_2_ and Fe^2+^. Excessive production of ROS can harm the mitochondrial respiratory chain, disturb mitochondrial membrane potential (ΔΨm), and impact Ca^2+^ balance. These free radicals are the main cause of oxidative damage involved in several diseases, including neurodegenerative diseases [66]. In our study, cardamom and fennel extracts provided neuroprotection against H_2_O_2_-induced oxidative stress in the cells. However, the pure compounds failed to do so. The neuroprotective potential observed from highest to lowest was cardamom-H > cardamom-EA > fennel-H > fennel-EA. The results suggest the synergistic activity of all other phytocompounds in the extracts for neuroprotective activity. The neuroprotective mechanism was further explored by measuring ROS and MMP.

Cardamom extracts restored the normal ROS levels at 30 µg/mL, whereas fennel-H accomplished the same effect at 1 µg/mL, suggesting that fennel-H has better ROS scavenging potential than cardamom in the SH-SY5Y cells. The decrease in ∆Ψm at higher concentrations by fennel-EA (30 μg/mL) and cardamom-H and fennel-H (10 and 30 μg/mL) indicated the incapability of the extracts in restoring MMP at those concentrations (Figure 8), even though they effectively ameliorated the ROS levels (Figure 7). Apart from the mitochondria, several other sources contribute to intracellular ROS in the cell. The reduced ability of some extracts at higher concentrations indicates their involvement in ROS scavenging activity from other cellular locations [27]. No doubt mitochondria are the chief generator of intracellular ROS; it is not the only source. Other cellular sources include enzymes (xanthine oxidase, lipoxygenase, cyclooxygenase, NADH/NADPH oxidase), peroxisomal β-oxidation of fatty acids, microsomal metabolism of xenobiotics, etc., which also contribute to ROS production. Hence, it appears that Cardamom-H and fennel-H can also scavenge ROS generated from other cellular sites.

A higher TPC could be the reason for the better ROS-nullifying activity of fennel extracts. Dietary polyphenols’ free radical capturing activity has a preventive role in oxidative stress-mediated diseases [67]. Fennel essential oils have been reported to reduce ROS and improve MMP in human liver cancer cells (Hep G2), human renal embryonic cells (HEK 293) [68], and human colon carcinoma cells (HCT116) [69]. Previously, α-terpinyl acetate has exhibited neuroprotective activity in H_2_O_2_-induced oxidative stress in (PC12) cells [18]. However, we did not get neuroprotection by α-terpinyl acetate in SH-SY5Y cell lines. Anethol has shown neuroprotection by affecting calcium homeostasis, lowering Aβ toxicity, and exerting antioxidant and anti-inflammatory effects in some reports [26,70]. Yet, we have not observed any neuroprotective effect with anethol in SH-SY5Y cell lines. Additional studies are required to elucidate the circumstances in which it may prove effective.

Pure compounds might not demonstrate bioactivity independently, yet they can display significant activity when found in an extract owing to the collaborative impacts of other phytocompounds present in the extract and the presence of co-factors, which might enhance the stability, bioavailability, and metabolism of the compound as well as shield it from degradation. In addition, biological pathways can be intricate and may necessitate several compounds working on various targets at the same time. A pure compound might not entirely activate the pathway, whereas the extract does. Pure compounds can non-specifically attach to proteins or other substances, which decreases their bioavailability. In the extract, various substances can diminish this non-specific attachment. In an extract, its optimal concentration may be naturally enhanced by the inclusion of additional components. The extracts provide a richer chemical environment that mimics natural synergy and enhances the bioactivity of their ingredients. This highlights the importance of studying both pure compounds and extracts in the quest for novel medications. It would be quite interesting to evaluate the effect of each compound. From the GC/MS results (Supplementary Information), there are over 10 compounds in each extract, out of which α-terpinyl acetate and anethol were present in the highest concentration. However, to thoroughly investigate all the bioactive compounds, considerable time and resources would be required.

The promising diversity of the bioactive compounds found in plant extracts combined with their relatively low cost and potential synergistic effects make them an attractive prospect for neuroprotection, but there are serious limitations related to variability in phytochemical composition (due to geographical location, variety, harvest time, climatic conditions, plant part used, storage, and extraction methods), lack of standardization of extraction methods, and challenges in isolating specific species or strains of compounds. Overcoming these challenges will result in more dependable and accurate studies on plant extracts for neuroprotective research.

## 4. Materials and Methods

### 4.1. Chemicals

Acetylcholinesterase (*Electrophorus electricus*, Type VI-S), anethol, ascorbic acid, 2,2′-azinobis-(3-ethylbenzothiazoline-6-sulfonic acid) (ABTS), aluminum chloride, acetyl thiocholine chloride (ATC), 2,2-diphenyl-1-picrylhydrazyl (DPPH), 5,5′-dithiobis (2-nitrobenzoic acid) (DTNB), 2′,7′-dichlorofluorescin diacetate (DCFDA), Folin–Ciocalteu reagent (FCR), ferrous sulfate (FeSO4), galantamine, gallic acid, hydrogen peroxide (H_2_O_2_), thioflavin T (ThT), 2,4,6-tripyridyl-s-triazine (TPTZ), tetramethylrhodamine, ethyl ester (TMRE), potassium persulfate, quercetin, and all organic solvents of HPLC grade were from Sigma-Aldrich (St. Louis, MO, USA). α-terpinyl acetate was purchased from Santa Cruz Biotechnology (Dallas, TX, USA). The WST-8 kit was purchased from Roche Diagnostics GmbH (Mannheim, Germany). Aβ1–42 was purchased from GenicBio Inc. (Shanghai, China), the purified anti-Aβ1–16 antibody was from Biolegend (San Diego, CA, USA), and the horseradish peroxidase (HRP)-conjugated W0–2 monoclonal antibody was procured from Peoplebio Inc. (Seongnam, Republic of Korea). 3,3′,5,5′-tetramethylbenzidine solution (TMB), fetal bovine serum (FBS), kanamycin, penicillin, and phosphate-buffered saline (PBST) were purchased from Thermo Fisher Scientific (Waltham, MA, USA). Dulbecco’s modified Eagle’s medium (DMEM) was supplied by Gibco (Thermo Fisher, Seoul, Republic of Korea).

### 4.2. Plant Material and Extraction

Cardamom (*Elettaria cardamomum* (L.) Maton) and Fennel (*Foeniculum vulgare* Mill.) seeds were bought from Expat Mart (Seoul, Republic of Korea). The dried seeds (25 g) were powdered and sequentially extracted using hexane and ethyl alcohol. The ratio of plant material to solvent was 1:10 (25 g sample in 250 mL solvent). Hexane was added to the conical flask containing the powdered samples and was subjected to shaking overnight (BioFree shaker, Seoul, Republic of Korea). The extraction procedure was performed twice and filtrated using Whatman No. 1 filter paper. Ethyl acetate was added to the remaining residue after air drying, and the extraction procedure was repeated. The solvent was evaporated by indirect heating using the water bath set on a heating plate (MTOPS MS300, Misung Scientific Equipment Co., Ltd., Seongnam-si, Gyeonggi-do, Republic of Korea). The fractions were weighed and stored until further experiments at 4 °C.

### 4.3. Gas Chromatography-Mass Spectrometry (GC-MS) Method

The GC-MS [5] was used for the identification of phytocompounds present in cardamom and fennel extracts. The samples were analyzed on a fused-silica capillary column (DB-5ms UI, 30 m × 0.25 mm i.d., film thickness 0.25 μm, Agilent, CA, USA) installed on GCMS-QP2020 (Shimadzu, Kyoto, Japan). The oven temperature was automated at 60 °C for 2 min, 100 °C at 4 °C/min, 290 °C at 10 °C/min, and finally isothermic for 10 min. The split injection mode (1:10) was used, and hexane and ethyl acetate fractions (1 μL, 1 mg/mL) were injected into the GC/MS via an auto-injector. The carrier gas was helium at a constant flow mode rate of 1 mL/min. The injection port, ion source, and interface temperatures were: 280, 280, and 150 °C, respectively. The energy of ionization was 70 eV. The mass spectra were obtained in full scan mode (40–700 AMU). The unknown phytocompounds were identified by a matching library of known compounds in the National Institute of Standards and Technology (NIST).

### 4.4. Determination of Total Phenolic Content (TPC) and Total Flavonoid Content (TFC)

Plants have a range of phytochemicals, among which phenolic and flavonoid compounds are important. Colorimetric methods were used to determine TPC and TFC. The reading was measured in the multimode-plate reader (Synergy-H1 BioTek, Agilent, Santa Clara, CA, USA). The TPC of extracts at 1 mg/mL concentration was measured using the FC reagent with modifications to the original method [71]. For calibration, gallic acid was used as standard (10–100 µg/mL). TPC was expressed as mg gallic acid equivalents (GAE) per g of extracts. The TFC of extracts at 1 mg/mL concentration was measured using an aluminum chloride-based assay with modifications to the original method [72]. Quercetin was used as standard (10–100 µg/mL) for calibration. TFC was expressed as mg quercetin equivalents (QE) per g of extract.

### 4.5. Antioxidant Capacity Determination

1. ABTS Radical Scavenging Assay

For antioxidant assays, 1 mg/mL of the sample (extracts/pure compound) was used. The free radical scavenging activity was measured using the multimode reader (SPECTROstar Nano, BMG Labtech, Ortenberg, Germany) after modifications to a previous method [73]. ABTS and potassium persulfate were mixed in equal volumes and kept in the dark at room temperature to produce ABTS radicals. After 30 min, the extracts were added and incubated again at room temperature for 30 min in the dark, and the absorbance value was determined at 734 nm. The percent radical scavenging activity (% RSA) was calculated using the equation:

% RSA = (Ab – As/Ab) × 100

where, Ab = absorbance of the blank, and As = absorbance of the sample.

2. DPPH Radical Scavenging Assay

For DPPH assays, 1 mg/mL of the sample (extracts/pure compound) was used as described in a previous method [74]. The samples were incubated with ethanolic DPPH at 120 µM concentration for 30 min at room temperature in the dark. Ascorbic acid (0.1–10 µg/mL) was used as a reference. The absorbance value was determined at 515 nm using the multimode reader (SPECTROstar Nano, BMG Labtech, Germany).% RSA was calculated as follows:

% RSA = (Ab − As/Ab) × 100

where, Ab = absorbance of the blank; As = absorbance of the sample.

3. FRAP Assay

For the experiment, the extracts and pure compounds were incubated with 200 µL of FRAP reagent for 30 min at room temperature [75]. For positive control, 2 µg/mL ascorbic acid was used, and the absorbance value was determined at 593 nm using the multimode reader (SPECTROstar Nano, BMG Labtech, Germany). The FRAP values are calculated from the FeSO_4_ (15–250 µM) standard curve and expressed as µM Fe^2+^/g.

### 4.6. Anti-Acetylcholinesterase Activity

AChE hydrolyses the neurotransmitter acetylcholine (ACh), leading to disruption of the cholinergic pathway in AD. Therefore, compounds displaying anti-AChE activity are of great relevance in AD. The anti-AChE activity was measured using Ellman’s method with slight modifications [76]. Cardamom and fennel extracts and pure compounds were incubated with AChE and 10 mM ATC for 15 min at 37 °C. DTNB (15 mM) was used to stop the reaction. The absorbance was determined using a multimode reader at 412 nm (SPECTROstar Nano, BMG Labtech, Germany). For a positive control, galantamine was used. The percent inhibition was computed using the equation:

Inhibition percentage (I%) = [(Ao − Ac) − (Ai − Aw)]/(Ao − Ac) × 100

where, Ao = absorbance without inhibitor; Ac = negative control; Ai = absorbance of inhibitor; Aw = negative control with inhibitor

### 4.7. Thioflavin T (ThT) Assay

The ThT assay is used to monitor amyloid aggregation. The compounds that inhibit Aβ self-assembly have therapeutic potential in NDs. The anti-Aβ1-42 fibrilization activity of extracts and their pure compounds was measured using a ThT assay [29]. The extracts and pure compounds were incubated at 37 °C in the presence/absence of 5 μM Aβ1-42. After 24 h of incubation, 100 µM ThT was added and incubated at 37 °C for approximately 15 min. The fluorescence intensity was determined at Ex 450 nm/Ems 490 nm using a multimode reader (Victor3, PerkinElmer, Shelton, CT, USA). Phenol red (50 µM) was used as positive control, and percent aggregation inhibition was computed using the equation:

Percent inhibition (I%) = [(1 − Fi/Fc) × 100]

where, Fi = fluorescence intensity with inhibitor; Fc = fluorescence intensity without inhibitor.

### 4.8. Anti-Aβ1-42 Oligomerization Activity

The anti-Aβ1-42 oligomerization activity of extracts and their pure compounds was measured using a Multimer Detection System (MDS) [1]. The extracts and their pure compounds were incubated along with 125 μg/mL Aβ1-42 for varied times (0, 2, and 4 h) at room temperature. The sample incubation was performed on pre-coated anti-β-amyloid plates to which HRP-conjugated W0-2 monoclonal antibody was added after 1 h and further incubated for 30 min at room temperature. TMB was added, and the absorbance value was determined after 15 min using a plate reader (Victor3, PerkinElmer, Shelton, CT, USA) at 450 nm.

### 4.9. Cell Culture

SH-SY5Y (ATCC CRL-2266, Manassas, VA, USA) human neuroblastoma cells were used for cell-based studies. The cells were maintained using DMEM enhanced with 10% FBS, 1% kanamycin, and 1% penicillin. For optimal growth, the cells were kept in a 95% humidified atmosphere at 37 °C in the incubator with 5% CO_2_. The experiments were performed after obtaining 70–80% confluency.

### 4.10. Cell Viability and Neuroprotection Assay

For cell viability and neuroprotection assay [1], cells were seeded in 96-well sterile plates at a concentration of 1 × 10^4^ cells/well and further incubated with samples at varied concentrations (µg/mL) for 24 h. For the cell viability assay, the samples were removed, and 10% WST-8 reagent was added for 2 h, and the absorbance value was determined at 450 nm using a multimode reader (Victor3, PerkinElmer, Shelton, CT, USA). The cytotoxicity percentage was computed using the equation:

Cytotoxicity% = [(Ac − At)/Ac] × 100

where Ac = absorbance of control cells; At = absorbance of treated cells.

For the neuroprotection assay, oxidative stress was induced by H_2_O_2_ in SH-SY5Y cells. The cells were preincubated with the samples for 24 h. The samples were removed, the cells were treated with H_2_O_2_ (100 µM) for 6 h, and later 10% WST-8 reagent was added for 2 h. H_2_O_2_ alone was used as solvent control, and the absorbance value was determined at 450 nm (Victor3, PerkinElmer, Shelton, CT, USA).

### 4.11. Measurement of Intracellular Reactive Oxygen Species (ROS)

The cells were seeded at 1 × 10^4^ cells/well concentration and incubated with the samples for 24 h. The samples were removed, and the cells were treated with H_2_O_2_ (100 µM) for 4 h to induce oxidative stress. H2DCFDA dye (25 µM) was added and incubated for 2 h in the dark at 37 °C. Fluorescence intensity was determined at Ex 495 nm/Ems 520 nm using a multimode reader (Victor3, PerkinElmer, Shelton, CT, USA). The ROS percentage was computed using the equation:

ROS% = [(Fc − Ft)/Fc] × 100

where, Fc = fluorescence of control cells; Ft = fluorescence of treated cells.

### 4.12. Mitochondrial Membrane Potential (ΔΨm) Assay

The cells were seeded at 1 × 10^4^ cells/well concentration and incubated with the samples for 24 h. The samples were removed, and the cells were treated with H_2_O_2_ for 2 h at 200 µM concentration [1]. The cells were incubated with TMRE dye (1 µM) for 1 h at 37 °C. Fluorescence intensity was determined at Ex 549 nm/Ems 575 nm using a multimode reader (Victor3, PerkinElmer, Shelton, CT, USA). ΔΨm percentage was computed using the equation:

ΔΨm% = [(Fc − Ft)/Fc] × 100

where, Fc = fluorescence of control cells; Ft = fluorescence of treated cells.

### 4.13. Data and Statistical Analysis

Data were presented as the mean ± SD of three sets of experiments. One-way ANOVA followed by Dunnett’s post hoc test was used for statistically analyzing the data. The symbols ####, **** represents *p* < 0.0001, ###, *** represents *p* <0.001, ##, ** represents *p* < 0.01, and #, * represents *p* < 0.05. The symbol # compares data significance from H_2_O_2_ control, whereas * compares data significance from untreated control. The IC_50_ values were determined using non-linear regression analysis (GraphPad Prism 10.2). The Vmax and Km were calculated using a Michaelis–Menten plot, and Lineweaver–Burk plots were drawn using linear regression analysis (GraphPad Prism 10.2).

## 5. Conclusions

The study provides phytochemical, acellular, and cell-based evidence to support the neuroprotective ability of the two natural mouth fresheners. Cardamom and fennel extracts provided significant neuroprotection in H_2_O_2_-induced oxidative stress in human neuroblastoma SH-SY5Y cell lines. The SH-SY5Y cell line is a human neuroblastoma cell line that can mimic certain neurodegenerative processes. It is easy to culture and is often used as a model to study neuroprotection. Fennel restored ROS levels to normal at a lower concentration (1 µg/mL), whereas cardamom did the same at 30 µg/mL. Both the samples restored MMP levels starting from 1 µg/mL. TPC and TFC were higher in fennel extract than cardamom extracts, which could be the reason for its better antioxidant activity. The pure compounds (α-terpinyl acetate and anethol) did not show neuroprotection in SH-SY5Y cells, suggesting the synergistic role of phytocompounds present in the extract. Extracts offer a more comprehensive chemical setting that replicates natural synergy and boosts the bioactivity of their components. This emphasizes the significance of examining both pure substances and extracts in the search for new drugs. Cardamom extracts exerted modest anti-AChE activity (IC_50_ 130–150 μg/mL) while α-terpinyl acetate showed good AChE inhibition (IC_50_ 61.87 μg/mL) and inhibited AChE by mixed-type pattern. The extracts exhibited strong anti-Aβ fibrilization activity (over 50%), but the pure compounds merely affected Aβ-fibrilization. Anethol showed a potent Aβ oligomerization inhibition, followed by α-terpinyl acetate and fennel-H. Overall, the extracts provided better antioxidant activity and neuroprotection than the pure compounds in cellular assays and displayed strong anti-Aβ fibrillization activity. Hence, the multifaceted neuroprotective approach shown by cardamom and fennel makes them appropriate candidates for drug development. Although the in vitro results appear promising, further in vivo and clinical studies are required to determine the optimal dosage of the plant extracts in NDs. Moreover, for studying specific neurodegenerative diseases (AD, PD), testing in other cell types may reveal more comprehensive and applicable information about human neuronal physiology and the effects of neuroprotective agents, which could make research results more significant.

## Figures and Tables

**Table 1:** Total phenolics, flavonoids, and antioxidant activity displayed by cardamom and fennel extracts and main bioactive compounds.

Extracts	Total Phenolic Content (mg GAE/g)	Total Flavonoid Content (mg QE/g)	FRAP (µM Fe^2+^/g)	DPPH (% RSA)	ABTS (% RSA)
Cardamom-H	7.95 ± 0.94	4.49 ± 1.01	15.07 ± 5.17	3.72 ± 0.0	15.10 ± 0.94
Cardamom-EA	10.21 ± 1.44	42.19 ± 0.94	2.23 ± 0.15	6.44 ± 0.0	17.55 ± 2.52
Fennel-H	26.91 ± 7.31	10.82 ± 0.80	64.70 ± 2.06	4.06 ± 0.0	6.58 ± 0.59
Fennel-EA α-Terpinyl acetate Anethol	32.43 ± 0.08	13.63 ± 0.50	76.78 ± 4.31 31.61 ± 1.17 59.11 ± 1.57	9.83 ± 0.47 4.01 ± 0.84 5.49 ± 1.89	24.59 ± 1.09 13.46 ± 0.01 15.87 ± 0.15
Positive control			230.56 ± 4.55 (10 μg/mL Ascorbic acid)	100 ± 0.35 (10 μg/mL Ascorbic acid)	97.61 ± 0.88 (10 μg/mL Quercetin)

ABTS: 2,2′-azinobis-(3-ethylbenzothiazoline-6-sulfonic acid; DPPH: 2,2-diphenyl-1-picrylhydrazyl; FRAP: ferric reducing antioxidant power; GAE: gallic acid equivalent; QE: quercetin equivalent; RSA: radical scavenging activity. Values were expressed as mean ± SD (*n* = 3).

**Table 2:** Kinetic data for AChE inhibition by cardamom extracts, α-terpinyl acetate, and anethol.

	Vmax (μmol/min/mg)	Km (mM)	Inhibition Type
No inhibitor	1.129	1.648	
Cardamom-H (100 μg/mL)	1.105	2.117	Mixed
Cardamom-H (200 μg/mL)	1.1033	2.595	
Cardamom-EA (100 μg/mL)	1.117	2.379	Mixed
Cardamom-EA (200 μg/mL)	1.176	3.821	
α-Terpinyl acetate (100 μg/mL)	1.082	4.887	Mixed
α-Terpinyl acetate (200 μg/mL)	1.034	7.882	
Anethol (100 μg/mL)	1.047	1.899	Mixed
Anethol (200 μg/mL)	1.041	2.258	

Km: Michaelis constant; Vmax: maximum velocity.

## Data Availability

Data are contained within the article. Additional information is provided in the Supplementary Materials.

## Supplementary Materials

The following supporting information can be downloaded at: https://www.mdpi.com/article/10.3390/ph18010002/s1, Figure S1: GC-MS Chromatograms of (A) Cardamom-H, (B) Cardamom-EA, (C) Fennel-H, and (D) Fennel-EA extract.
