# Supplementary material for: Multivalent Neuroprotective Activity of Elettaria cardamomum (Cardamom) and Foeniculum vulgare (Fennel) in H2O2-Induced Oxidative Stress in SH-SY5Y Cells and Acellular Assays
Source: Pharmaceuticals (Basel). 2024 Dec 24;18(1):2. doi: 10.3390/ph18010002 (PMC11768314; doi:10.3390/ph18010002)
Supplement: Supplementary file 1 [file pharmaceuticals-18-00002-s001.zip › pharmaceuticals-3362146-supplementary.pdf]

Supplementary Information

Multivalent neuroprotective activity of Elettaria cardamomum (Cardamom) and Foeniculum vulgare (Fennel) in H2O2-induced oxidative stress in SH-SY5Y cells and acellular assays

Figure S1: GC-MS Chromatograms of (A) Cardamom-H, (B) Cardamom-EA, (C) Fennel-H, and (D) Fennel-EA extracts.

A

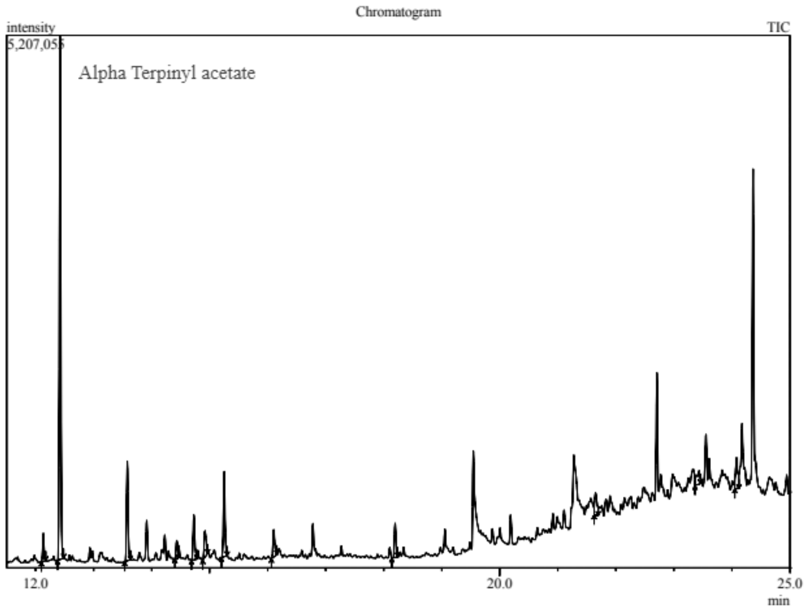

Peak Report TIC

R.Time	Area	Area%	Height	Height%	Name
12.134	455497	2.57	274813	2.94	Limonene oxide, cis-
12.422	8909289	50.36	5125035	54.89	.alpha.-Terpinyl acetate
13.580	1758586	9.94	976137	10.45	2-((1R,4R)-4-Hydroxy-4-methylcyclohex-2-[illegible]
14.433	359177	2.03	177888	1.91	Hydroxy-.alpha.-terpenyl acetate
14.726	926244	5.24	440951	4.72	Sobrerol 8-acetate
14.919	603209	3.41	255668	2.74	Hydroxy-.alpha.-terpenyl acetate
15.250	1547198	8.75	833442	8.93	8-Acetoxycarvotanacetone
16.102	578966	3.27	267669	2.87	2-Oxabicyclo[2.2.2]octan-6-ol, 1,3,3-trimeth[illegible]
18.196	602331	3.40	335120	3.59	Ambrial
21.650	611253	3.45	220543	2.36	(2-(1,3-Dioxolan-2-yl)ethyl)triphenylphosph[illegible]
23.440	525283	2.97	125461	1.34	(E)-Labda-8(17),12-diene-15,16-dial
24.081	815003	4.61	304881	3.27	1,2-Propanediol, 3-benzyloxy-1,2-diacetyl-
	17692036	100.00	9337608	100.00	

B.

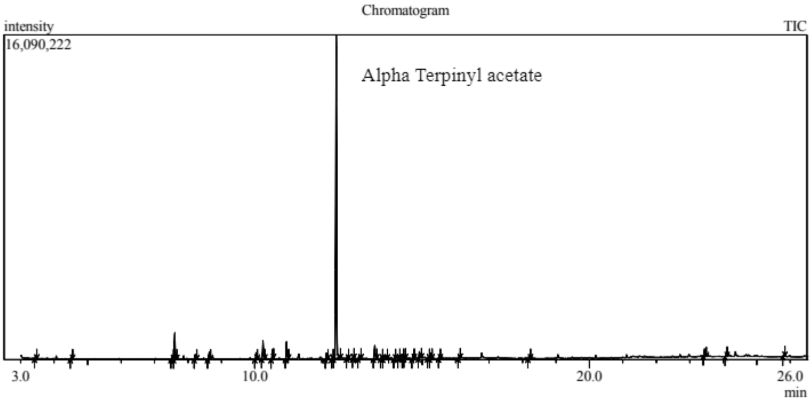

Peak Report TIC

R.Time	Area	Area%	Height	Height%	Name
3.440	176303	0.38	94600	0.38	5-Chloropentanoic acid, 2-tetrahydrofurylme
4.512	245796	0.53	141103	0.57	Benzene, chloro-
7.527	345955	0.75	203122	0.82	D-Limonene
7.604	2233091	4.84	1295393	5.24	Eucalyptol
8.218	419926	0.91	233174	0.94	5-Isopropyl-2-methylbicyclo[3.1.0]hexan-2-
8.635	632628	1.37	334782	1.35	Linalool
10.036	525103	1.14	310550	1.26	3-Cyclohexen-1-ol, 4-methyl-1-(1-methyleth
10.241	1523532	3.30	897215	3.63	L-.alpha.-Terpineol
10.523	735762	1.59	451244	1.83	.gamma.-Terpinene
10.946	1462943	3.17	842362	3.41	Linalyl acetate
12.140	493197	1.07	295925	1.20	Limonene oxide, cis-
12.317	101533	0.22	63202	0.26	Cyclohexanol, 1-methyl-4-(1-methylethenyl)-
12.431	30520288	66.09	16022385	64.83	.alpha.-Terpinyl acetate
12.766	254573	0.55	161811	0.65	Geranyl acetate
12.939	184930	0.40	128086	0.52	2,6,10-Dodecatrienal, 3,7,11-trimethyl-, (Z,E
13.110	360594	0.78	120510	0.49	1,7-Octadiene-3,6-diol, 2,6-dimethyl-
13.583	1247933	2.70	658527	2.66	2-((1R,4R)-4-Hydroxy-4-methylcyclohex-2-
13.788	223444	0.48	122912	0.50	Limonene oxide, cis-
13.918	327934	0.71	149115	0.60	2-((1R,4R)-4-Hydroxy-4-methylcyclohex-2-
14.180	179941	0.39	74729	0.30	2-Octen-1-ol, 3,7-dimethyl-, isobutyrate, (Z)-
14.295	131436	0.28	83539	0.34	.delta.-Terpineol, acetate
14.432	239079	0.52	148979	0.60	Hydroxy-.alpha.-terpenyl acetate
14.471	180735	0.39	111553	0.45	.alpha.-Guaiene
14.726	386350	0.84	218751	0.89	Humulane-1,6-dien-3-ol
14.916	818998	1.77	323209	1.31	Hydroxy-.alpha.-terpenyl acetate
15.188	128366	0.28	81911	0.33	1,6,10-Dodecatrien-3-ol, 3,7,11-trimethyl-, (
15.252	501864	1.09	271597	1.10	8-Acetoxycarvotanacetone
15.513	100222	0.22	62025	0.25	2,2,4-Trimethyl-1,3-pentanediol diisobutyrat
16.104	173101	0.37	96884	0.39	exo-2-Hydroxycineole
18.199	260478	0.56	146710	0.59	Ambrial
23.442	674044	1.46	359380	1.45	(E)-Labda-8(17),12-diene-15,16-dial
24.084	232564	0.50	130663	0.53	1,2-Propanediol, 3-benzyloxy-1,2-diacetyl-
25.814	159609	0.35	79835	0.32	(E)-15,16-Dinorlabda-8(17),12-dien-14-al
	46182252	100.00	24715783	100.00	

(C)

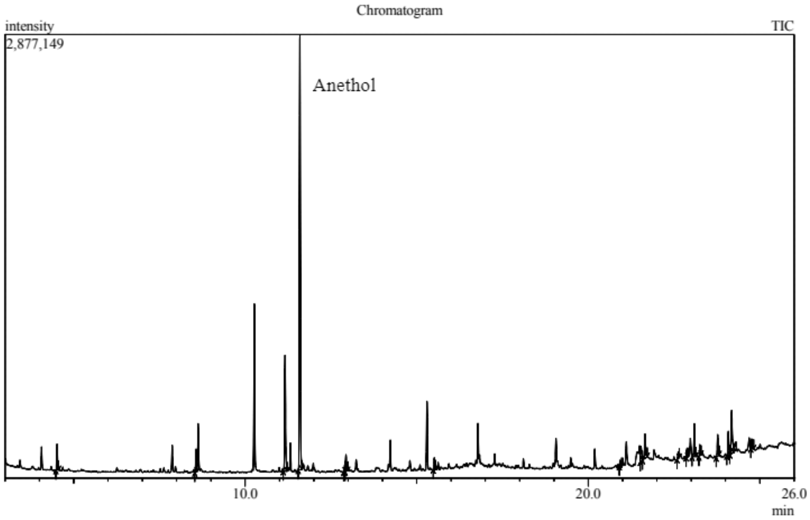

Peak Report TIC

R.Time	Area	Area%	Height	Height%	Name
4.515	282737	2.73	168371	3.10	Benzene, chloro-
8.569	259828	2.51	148791	2.74	L-Fenchone
11.161	1466555	14.17	751849	13.83	Benzaldehyde, 4-methoxy-
11.593	5132867	49.59	2822364	51.90	Anethole
12.895	82627	0.80	41000	0.75	Benzene, (1-methoxyethyl)-
12.936	207889	2.01	108835	2.00	2-Propanone, 1-(4-methoxyphenyl)-
15.513	127835	1.23	76740	1.41	2,2,4-Trimethyl-1,3-pentanediol diisobutyrat
20.934	72960	0.70	41589	0.76	9-Octadecenoic acid, methyl ester, (E)-
21.540	241799	2.34	93456	1.72	(Z)-Ethyl heptadec-9-enoate
21.654	395158	3.82	160121	2.94	Vinyltriphenylphosphonium bromide
22.621	76252	0.74	44992	0.83	Citronellyl palmitoleate
22.875	92616	0.89	55453	1.02	Octadecanoic acid, 6-hydroxy-, methyl ester
23.090	394608	3.81	214599	3.95	2H-Pyran, 2-(2-heptadecynyloxy)tetrahydro-
23.252	125666	1.21	72751	1.34	1,2-Oxathiane, 6-dodecyl-, 2,2-dioxide
23.774	261311	2.52	139569	2.57	9-Octadecenoic acid, 1,2,3-propanetriyl ester,
24.084	289148	2.79	153572	2.82	1,2-Propanediol, 3-benzyloxy-1,2-diacetyl-
24.180	710020	6.86	281749	5.18	Glycidyl oleate
24.762	131532	1.27	61848	1.14	Triphenylphosphine oxide
	10351408	100.00	5437649	100.00	

(D)

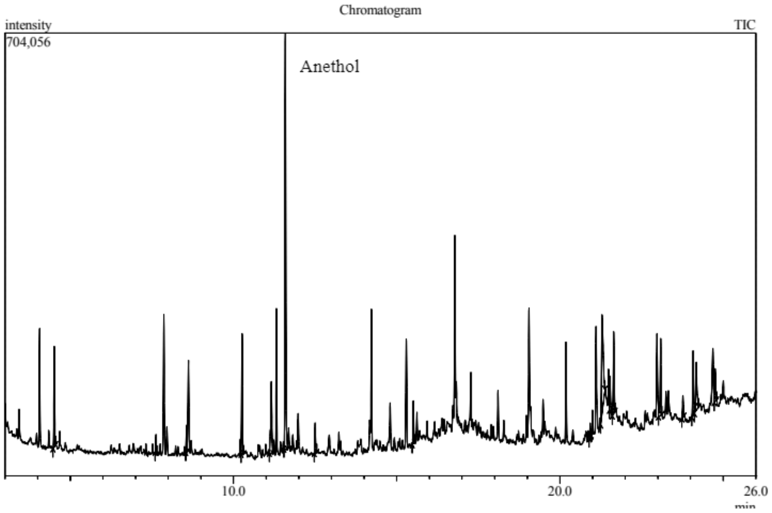

Peak Report TIC

R.Time	Area	Area%	Height	Height%	Name
4.509	273552	6.53	160016	7.71	Benzene, chloro-
7.626	53993	1.29	29352	1.41	Benzene, 1,2-dichloro-
8.563	73190	1.75	42899	2.07	L-Fenchone
10.262	338031	8.06	191534	9.23	Anethole
11.158	279725	6.67	114528	5.52	Benzaldehyde, 4-methoxy-
11.588	1218807	29.08	661292	31.87	Anethole
12.502	87510	2.09	47764	2.30	Eugenol
15.509	110728	2.64	68252	3.29	2,2,4-Trimethyl-1,3-pentanediol diisobutyrat
20.926	44826	1.07	26607	1.28	6-Octadecenoic acid, methyl ester, (Z)-
21.290	568451	13.56	157549	7.59	cis-9-Hexadecenal
21.530	93682	2.23	55437	2.67	(Z)-Ethyl heptadec-9-enoate
21.647	256032	6.11	130631	6.30	Vinyltriphenylphosphonium bromide
23.085	225387	5.38	122591	5.91	2H-Pyran, 2-(2-heptadecynyloxy)tetrahydro-
23.768	59328	1.42	34809	1.68	Oleic anhydride
24.080	205927	4.91	102198	4.93	1,2-Propanediol, 3-benzyloxy-1,2-diacetyl-
24.175	200401	4.78	76870	3.71	Glycidyl oleate
24.755	102047	2.43	52332	2.52	Triphenylphosphine oxide
	4191617	100.00	2074661	100.00	
